# Validity of Self-Reported Tobacco Smoke Exposure among Non-Smoking Adult Public Housing Residents

**DOI:** 10.1371/journal.pone.0155024

**Published:** 2016-05-12

**Authors:** Shona C. Fang, Shan Chen, Felicia Trachtenberg, Slawa Rokicki, Gary Adamkiewicz, Douglas E. Levy

**Affiliations:** 1 New England Research Institutes, Inc., Watertown, MA, United States of America; 2 Interfaculty Initiative in Health Policy, Harvard University, Cambridge, MA, United States of America; 3 Department of Environmental Health, Harvard T.H. Chan School of Public Health, Boston, MA, United States of America; 4 Mongan Institute for Health Policy, Massachusetts General Hospital, Harvard Medical School, Boston, MA, United States of America; 5 Tobacco Research and Treatment Center, Massachusetts General Hospital, Boston, MA, United States of America; Geisel School of Medicine at Dartmouth College, UNITED STATES

## Abstract

**Introduction:**

Tobacco smoke exposure (TSE) in public multi-unit housing (MUH) is of concern. However, the validity of self-reports for determining TSE among non-smoking residents in such housing is unclear.

**Methods:**

We analyzed data from 285 non-smoking public MUH residents living in non-smoking households in the Boston area. Participants were interviewed about personal TSE in various locations in the past 7 days and completed a diary of home TSE for 7 days. Self-reported TSE was validated against measurable saliva cotinine (lower limit of detection (LOD) 0.02 ng/ml) and airborne apartment nicotine (LOD 5 ng). Correlations, estimates of inter-measure agreement, and logistic regression assessed associations between self-reported TSE items and measurable cotinine and nicotine.

**Results:**

Cotinine and nicotine levels were low in this sample (median = 0.026 ng/ml and 0.022 μg/m^3^, respectively). Prevalence of detectable personal TSE was 66.3% via self-report and 57.0% via measurable cotinine (median concentration among those with cotinine>LOD: 0.057 ng/ml), with poor agreement (kappa = 0.06; sensitivity = 68.9%; specificity = 37.1%). TSE in the home, car, and other peoples’ homes was weakly associated with cotinine levels (Spearman correlations r_s_ = 0.15–0.25), while TSE in public places was not associated with cotinine. Among those with airborne nicotine and daily diary data (n = 161), a smaller proportion had household TSE via self-report (41.6%) compared with measurable airborne nicotine (53.4%) (median concentration among those with nicotine>LOD: 0.04 μg/m^3^) (kappa = 0.09, sensitivity = 46.5%, specificity = 62.7%).

**Conclusions:**

Self-report alone was not adequate to identify individuals with TSE, as 31% with measurable cotinine and 53% with measurable nicotine did not report TSE. Self-report of TSE in private indoor spaces outside the home was most associated with measurable cotinine in this low-income non-smoking population.

## Introduction

With the rate of tobacco smoking remaining unacceptably high in the US at 16.8% of adults [1], involuntary tobacco smoke exposure (TSE) continues to threaten the health of non-smokers [2, 3]. Risk of TSE is not equal in the population however, with some sub-populations, such as residents of multiunit housing (MUH), facing greater risk of involuntary exposure due to the ability of tobacco smoke to diffuse across units within MUH [4–6]. Surveys, biomarker studies, and environmental studies indicate the rate of tobacco smoke infiltration in MUH is high [7]. For example, 44–53% of MUH residents with smoke-free home rules reported experiencing tobacco smoke infiltration in their living unit that originated from elsewhere in or around their building [8–10]. Among children living in homes with non-smokers, those living in MUH have higher levels of cotinine, a common biomarker of TSE, than those living in detached housing (geometric mean 0.075 μg/m^3^; 95% CI: (0.062–0.091) vs. 0.031 μg/m^3^ (0.026–0.038)) [11]. Environmental studies indicate that the rates of infiltration may be even higher, with detectable levels of nicotine found in 89% of a sample of 19 smoke-free MUH homes (median 0.04 μg/m^3^; <LOD to 0.28) [12]. Given the large number of people in the US living in MUH, the burden of TSE in MUH residents is substantial, with recent data estimating 27.6–28.9 million MUH residents with smoke-free home rules having tobacco smoke infiltration in their homes [13].

Among low-income residents living in public MUH, TSE is particularly high, likely related to the fact that smoking rates in low-income populations are higher than in populations of higher socioeconomic status.[14, 15] In November 2015, the U.S. Department of Housing and Urban Development Office of Public and Indian Housing issued a proposed rule to prohibit tobacco smoking in all federally subsidized units in publicly owned housing [16]. This position is motivated by the goal of protecting the health of non-smoking residents and reducing costs associated with cleaning units. Currently, approximately 500 of the 3,300 public housing authorities nationally have adopted smoke-free policies for some or all apartment buildings [16].

The ability to assess TSE with both accuracy and efficiency is critical for aiding stakeholders in understanding the burden of TSE among public and low-income housing residents living in MUH, and the impact of smoke-free policies on reducing TSE in non-smoking residents. More generally, accurately assessing TSE is important for public health planning, epidemiologic research, and clinical guidance. Biomarkers of TSE such as cotinine may be reliably quantified in urine, serum, hair, or saliva [17]. Cotinine is the primary metabolite of nicotine and is considered to be the best available biomarker of TSE; with a half-life of 16–20 hours, it reflects exposure over a period of 2–3 days [18]. Environmental samples of air or surfaces may also be used to monitor TSE. For example, both airborne nicotine and fine particulate matter are commonly measured as indicators of both in-unit smoking activity and between-unit transfer. Biological and environmental monitoring can be costly and inconvenient however, particularly in large populations. Instead, self-reports are often employed to assess TSE, either via interviewer- or self-administered questionnaires. Self-reported assessments of TSE have been validated against objective measures in general, working, pediatric, and certain patient populations, as well as in pregnant women, as reviewed by Avila-Tang et. al [17]. Little is known however about the validity of self-reported TSE in low-income residents living in MUH, whose sources and patterns of exposure may differ from other populations. In September 2012, the Boston Housing Authority (BHA), home to 25,000 residents, became the largest housing authority at the time to implement a comprehensive smoke-free public housing initiative. Using data from a study assessing the impact of the BHA smoking ban on non-smoking residents’ TSE (the FreshAir Study), we conducted a secondary analysis comparing self-reported personal and apartment TSE to saliva cotinine and apartment air nicotine with the goal of establishing whether self-reported TSE is a useful proxy for objectively-measured TSE among non-smoking individuals. As reports suggest no safe level of exposure to tobacco smoke and saliva cotinine is detectable at very low levels, we focused our comparison of self-reported TSE with the presence or absence of detectable saliva cotinine [2, 19].

## Materials and Methods

### Study design and population

The FreshAir Study was a prospective, quasi-experimental study designed to assess the impact of the BHA smoke-free policy on non-smoking residents’ TSE. The study was conducted in family housing developments of the BHA and Cambridge Housing Authority (CHA) in Massachusetts, with the CHA as a comparison non-intervention site. Participants were adult English- and Spanish-speaking non-smokers living in units where no other residents smoked (defined as current smokers of cigarettes, cigars, blunts, or pipes). We recruited one resident per household for the study. Residents using nicotine replacement therapies or other tobacco products and those living in townhouses were ineligible to participate.

We enrolled a convenience sample of 297 residents (199 from BHA and 98 CHA) into the study and interviewed participants about demographics and TSE inside and outside the building. Participants provided a saliva sample and had a passive nicotine monitor placed in the main living area of their apartment. Data for the current analyses were restricted to baseline data (June 2012 to October 2012, with October data for CHA only), before implementation of the smoke-free policy in BHA, to avoid any influence of the non-smoking policy on participant responses. Participants received $30 for completing baseline data collection. The study was approved by the Partners HealthCare and New England Research Institutes, Inc. Institutional Review Boards and participants provided informed consent.

### Saliva cotinine

A saliva sample of at least 4 ml was collected from each participant into a 20 ml polyethylene vial. Samples were stored on ice in the field and transferred to -20°C freezers at the end of the day. The Clinical Pharmacology Laboratory at the University of California San Francisco performed high sensitivity analysis of cotinine in saliva using liquid chromatography-tandem mass spectrometry (LC-MS/MS). The lower limit of detection (LOD) was 0.02 ng/ml saliva [20]. Measurable cotinine was defined by a value above the LOD. Twelve individuals were excluded from the analysis: two with insufficient saliva samples for analysis and ten deemed to be current smokers based on cotinine >15 ng/ml [21].

### Airborne nicotine

Airborne nicotine in participants’ homes was assessed using passive monitors developed at the University of California, Berkeley [22]. We deployed air monitors in the main living area of the home following each interview. Monitors were left in place unsealed for at least 7 days at which time they were collected and resealed by the field staff. Monitors were then stored sealed at room temperature until the end of the study when they were analyzed by gas chromatography at the University of California, Berkeley. The lower LOD was 5 ng. Measurable nicotine was defined by a value above the LOD. Each participant was assigned an apartment nicotine concentration, determined by dividing the nicotine mass by an effective sampling rate of 24 ml/minute that was multiplied by the total duration of deployment. Nicotine concentrations are reported as μg/m^3^.

### Self-reported TSE

A set of questions to assess TSE in and around the home was developed with input from housing authority residents, and piloted prior to the study. During interview, participants were asked about the presence and frequency of TSE in the home and car (yes/no, and if yes, number of days in past 7 days), as well as the relative frequency of TSE in the past 7 days at: the place of work/volunteer, bus or train stop, someone else’s home, a public area inside the building, outside the doorway of the building, and in another area outside the building (see Table 1 for specific questions). Relative frequency responses were on a 5-point Likert scale (never, rarely, sometimes, often, and all the time).

**Table 1:** Characteristics of study population (n = 285).

	No. or mean/median	Percent or range
Age in years, mean (range)	49.4	19- >85
Housing site		
Cambridge	95	33.3
Boston	190	66.7
Gender		
Male	51	17.9
Female	234	82.1
Race/ethnicity		
Non-Hispanic White	20	7.0
Non-Hispanic Black	96	33.7
Hispanic	148	51.9
Other	20	7.0
Speak English well or very well	181	63.5
Speak Spanish at home	144	50.5
Born in US	95	33.3
Education		
High school or less	189	66.3
Some college or associate degree	71	24.9
College or above	21	7.4
Marital status		
Married or living with a partner	90	31.6
Divorced, separated or widowed	84	29.5
Single, never married	111	38.9
Employed or full time student	124	43.5
Heath in general		
Very good/excellent	107	37.5
Good	89	31.2
Fair or poor	89	31.2
Measurable cotinine	161	56.5
Cotinine level > LOD (ng/ml), median (range)	0.057	0.021–10.96
Measurable apartment airborne nicotine^1^	86	53.4
Nicotine level >LOD (μg/m^3^) median (range)	0.042	0.021–1.34

^1^Among 161 samplers deployed for 7 days

In addition, participants reported household TSE prospectively over the course of 7 days using a daily diary. This specifically asked about TSE inside the apartment during the time the air monitor sampled for nicotine. For each of the seven days, participants were asked “Did anyone smoke tobacco anywhere in your home” and “Did you smell tobacco in your home?”.

### Statistical analysis

In order to create an overall indicator of self-reported TSE based on the interview items, we considered a score based on summing original responses on the interview items (3 items that asked about the number of days of exposure (0–7) and 7 items about the relative frequency of TSE on a 5-point Likert scale (1–5)). In the absence of time activity patterns (e.g. amount of time spent in different microenvironments such as work, bus stop, home) needed to appropriately weight items, each item was given equal weight. A receiver operating curve (ROC) analyses was conducted to identify an appropriate cutoff using measureable saliva cotinine as the gold standard. The area under the curve (AUC) was computed from the ROC; an AUC of 0.50 reflects a measure that cannot distinguish two groups any better than chance while an AUC of 1.0 indicates a perfect ability to discriminate.[23] The AUC was close to 0.50, suggesting no optimal cutoff or utility of this summary score for classifying individuals as having TSE or no TSE. We thus created an overall indicator of TSE (yes/no) based on a positive response to any of the self-reported TSE items (yes to presence of smoking in the home, smelling smoke in the home, or riding in the car where somebody had smoked; or ≥often/all of the time for Likert scale items). We compared the agreement (kappa statistic) of the overall TSE indicator based on self-report with presence or absence of measurable saliva cotinine. The sensitivity and specificity of self-reported personal TSE were calculated using saliva cotinine as the gold standard. A similar analysis was performed to validate self-reported household TSE obtained from the daily diary against measureable airborne nicotine in the apartment.

In order to determine the strength of monotonic associations between days of self-reported TSE items as captured in the interview and daily diary, and levels of objective measures (saliva cotinine and airborne nicotine), we used spearman correlations. To determine associations between dichotomous self-reported TSE items (yes/no) and measurable cotinine and nicotine, we constructed separate logistic regression models for each outcome. Likert scale items were dichotomized, with a response of often/all of the time reflecting positive TSE. Univariate models were constructed, as well as multivariable models mutually adjusted for each TSE item.

In sub-analysis to better understand distinct sources of exposure, we conducted a variable cluster analysis of the interview items measuring TSE in the past seven days using PROC VARCLUS in SAS. A goal of variable cluster analysis, similar to factor analysis, is to identify subgroups of items which are correlated with one another, in order to identify potential underlying dimensions in the questions. Each cluster represents items that are more similar to each other than to those in other clusters. While items may contribute to more than one scale in a factor analysis, and not all items may be used, the variable cluster analysis creates distinct, non-overlapping clusters using all items. For measurable cotinine, we constructed logistic regression models using the clusters that emerged from the variable cluster analysis. Statistical significance for all testing was established at the α = 0.05 level. Analyses were conducted in SAS 9.3 (SAS Institute, Cary, NC).

## Results

A total of 285 participants were included in the analysis of self-reported TSE and saliva cotinine. Participants were predominantly female and black or Hispanic with a mean age of 49 years (range 19–>85) (Table 1). One hundred-sixty-one participants (56.5%) had measureable saliva cotinine (median concentration among those with cotinine >LOD: 0.057 ng/ml; range: 0.021–10.96). The overall median (range) cotinine concentration for the entire study sample was 0.026 ng/ml (≤LOD–10.96). For the comparison of airborne nicotine measurements with the daily diary of TSE inside the home, we included only participants whose air sampler was deployed for exactly seven days (n = 161) (due to practical limitations, some samplers were deployed less than or more than 7 days). Of the 161 participants, similarly more than half (53.4%) had measurable nicotine in the apartment (median concentration among those with nicotine >LOD: 0.04 μg/m^3;^ range: 0.021–1.34). The overall median (range) nicotine concentration for the 161 subjects was 0.022 μg/m^3^ (≤LOD– 1.34).

### Personal TSE

The majority of the 285 participants (66.3%) reported personal TSE in the previous week in at least one location (“any self-reported personal TSE”). Smelling smoke outside the doorway of the building was most commonly reported (36.1%), followed by a public area inside the building (30.9%), another outside area of the building (30.2%), and in the home (29.5%) (Table 2). Median cotinine level was highest among those who reported anyone smoking in the home (n = 10, 0.155 ng/ml), while cotinine among those who reported exposure at the bus/train stop, a public area inside the building, outside the doorway of the building, or another outside area of the building was similar to those without any self-reported exposure (0.024 ng/ml).

**Table 2:** Distribution of saliva cotinine and apartment nicotine levels by self-reported TSE.

	**Interview response (n = 285)**^3^										** **
	**Yes**					**No**					** **
	** **	** **	**Cotinine (ng/ml)**			** **	** **	**Cotinine (ng/ml)**			** **
**Interview item:**	**No.**	**Percent**	**Min**	**Median**	**Max**	**No.**	**Percent**	**Min**	**Median**	**Max**	**p-value**†
Any self-reported personal TSE below^1^:	189	66.3	≤LOD	0.03	11	95	33.3	≤LOD	0.024	4.93	0.28
Outside doorway building*	103	36.1	≤LOD	0.025	3.11	182	63.9	≤LOD	0.029	11	0.13
Public area inside building*	88	30.9	≤LOD	0.021	2.17	197	69.1	≤LOD	0.03	11	0.09
Another outside area of building*	86	30.2	≤LOD	0.024	6.31	199	69.8	≤LOD	0.028	11	0.32
Smelled smoke in home at any time	84	29.5	≤LOD	0.03	7.17	201	70.5	≤LOD	0.026	11	0.38
Bus stop*	81	28.4	≤LOD	0.025	2.17	204	71.6	≤LOD	0.029	11	0.47
Someone else's home*	52	18.2	≤LOD	0.046	6.31	233	81.8	≤LOD	0.022	11	<0.001
Work*	34	11.9	≤LOD	0.028	7.17	251	88.1	≤LOD	0.026	11	0.32
Anyone smoked in car^2^	19	6.7	≤LOD	0.032	11	264	92.6	≤LOD	0.026	7.17	0.23
Anyone smoked in home	10	3.5	≤LOD	0.155	11	275	96.5	≤LOD	0.026	7.17	**0.01**
	**Daily diary response (n = 161)**^4^										** **
	**Yes**					**No**					** **
			**Nicotine (μg/m**^**3**^**)**					**Nicotine (μg/m**^**3**^**)**			** **
**Daily diary item:**	**No.**	**Percent**	**Min**	**Median**	**Max**	**No.**	**Percent**	**Min**	**Median**	**Max**	**p-value**†
Any TSE in the home below:	67	41.6	≤LOD	0.028	1.34	94	58.4	≤LOD	0.01	0.433	0.28
Smelled smoke in home	67	41.6	≤LOD	0.027	1.34	94	58.4	≤LOD	0	0.433	0.16
Anyone smoked in home	13	8.1	≤LOD	0.029	1.34	148	91.9	≤LOD	0.022	0.591	0.17

*Smelled smoke often/all of the time in location

^1^1 observation missing

^2^2 observations missing

^3^past 7 days

^4^ over 7 days

**†**Wilcoxon two-sample test p-value comparing cotinine (or nicotine) distributions by self-reported exposure (y/n)

Based on measurable cotinine, 161 (56.5%) were exposed, compared to the 66.3% from the self-reported index. Agreement between “any self-reported personal TSE” and measurable saliva cotinine was low (kappa = 0.06) (Table 3). The median (range) cotinine level among those with any self-reported personal TSE was 0.030 (<LOD– 10.96) vs. 0.024 (<LOD– 4.93) among those without self-reported TSE (Table 2; Wilcoxon two-sample test p-value = 0.28). Using cotinine as the gold standard, of the 161 individuals with measurable cotinine, 68.9% were concordantly classified as exposed based on self-report of any TSE in the past week (i.e. sensitivity or true positives), leaving 31.1% of individuals with measurable saliva cotinine classified as unexposed based on self-report (false negatives) (Table 3). Median measurable cotinine concentrations were similar among the true positives and false negatives (0.059 vs. 0.054 ng/ml), though the maximum exposure was substantially higher among true positives (10.96 vs. 4.93 ng/ml). Among individuals without measurable cotinine, 37.1% were concordantly classified as unexposed based on self-report (i.e. specificity or true negatives), while 62.9% reported TSE in the absence of measurable cotinine (false positives).

**Table 3:** Agreement between self-reported TSE and measurable cotinine and airborne nicotine.

	**Measurable cotinine (n = 161)**		**Non-detectable cotinine (n = 124)**					**Measurable cotinine concentration, above LOD (ng/ml)**	
**Interview:**	**No.**	**%**	**No.**	**%**	**Kappa**	**Sensitivity**	**Specificity**	**Median**	**Range**
Any personal TSE	111	68.9	78	62.9	0.06	68.9	37.1	0.059	0.021–10.96
No personal TSE	50	31.1	46	37.1				0.054	0.023–4.93
Total	161	100	124	100				0.057	0.021–10.96
	**Measurable nicotine (n = 86)**		**Non-detectable nicotine (n = 75)**					**Measurable nicotine concentration, above LOD (μg/m**^**3**^**)**	
**Daily diary:**	**No.**	**%**	**No.**	**%**	**Kappa**	**Sensitivity**	**Specificity**	**Median**	**Range**
Any TSE in the home	39	45.3	28	37.3	0.09	46.5	62.7	0.042	0.021–1.34
No TSE in the home	47	54.7	47	62.7				0.042	0.021–0.43
Total	86	100	75	100				0.042	0.021–1.34

Cotinine (ng/ml) was weakly though significantly correlated with days anyone smoked in the home (r_s_ = 0.25), days riding in a car in which somebody smoked (r_s_ = 0.16), and relative frequency of smelling smoke in someone else’s home outside the building (r_s_ = 0.15). Correlations between cotinine level and frequency of TSE in all other locations were weak and not statistically significant (data not shown). In logistic regression models, smelling smoke in other people’s homes in the past week was significantly associated with measurable cotinine in unadjusted and adjusted models (adjusted OR = 7.26; 95% CI: 3.1–17.2). Other self-reported TSE items were not associated with measurable cotinine (Table 4).

**Table 4:** Unadjusted and multivariable associations between self-reported TSE, measurable saliva cotinine, and airborne apartment nicotine.

	**Saliva cotinine**			
	**Univariate**		**Multivariable**	
**Self-reported personal TSE (y/n):**	**OR**	**95% CI**	**OR**	**95% CI**
Anyone smoked in the home	3.2	0.7–15.3	3.7	0.7–20.2
Anyone smoked in the car	1.1	0.4–2.7	0.7	0.2–2.0
Smelled smoke at/in:				
The home	1.1	0.7–1.9	1.1	0.5–2.0
Work/place of volunteer	1.5	0.7–3.1	1.4	0.6–3.1
Bus or train stop	0.9	0.5–1.5	0.8	0.4–1.5
Someone else's home	4.7*	2.2–10.0	7.3*	3.1–17.2
Public area inside building	0.7	0.4–1.2	0.5	0.3–1.1
Outside doorway building	0.9	0.5–1.4	0.9	0.5–1.7
Another outside area of building	0.8	0.5–1.4	0.8	0.4–1.7
	**Airborne apartment nicotine**			
	**Univariate**		**Multivariable**	
**Self-reported TSE in the home (y/n):**	**OR**	**95% CI**	**OR**	**95% CI**
Someone smoked in the home	1.4	0.5–4.6	1.2	0.4–4.1
Smelled tobacco smoke in the home	1.4	0.7–2.6	1.4	0.7–2.6

*p<0.05

Cluster analysis revealed three separate clusters: 1) “out of home—public” (e.g. bus/train stop, public areas inside the building); 2) “out of home—private” (work and other people’s homes); and 3) “personal spaces” (home and car). The first cluster accounted for 31.1% of the variability in the self-reported data, while all three components together explained 55.1% of the variation of the original variables. In logistic regression models, a summary score based on items in “out of home–private” was associated with measurable cotinine in both a univariate model, and a model mutually adjusted for other factors (OR = 1.2; 95% CI: 1.1–1.3), while the other clusters were not significantly associated with cotinine.

### Household TSE

The two items from the daily diary were collapsed into a single binary variable, “household TSE” (yes if on any of the 7 days the participant reported smelling smoke in the unit or somebody smoking in the unit). Sixty-seven (42%) reported household TSE (Table 2), with the majority having smelled smoke in the home, and few having had anyone smoke in the home (n = 13). Median (range) nicotine concentrations were 0.028 μg/m^3^ (≤LOD-1.34) among those with self-reported household TSE versus 0.010 μg/m^3^ (≤LOD-0.43) among those who reported no household TSE (Wilcoxon two-sample test p-value = 0.17).

Agreement between household TSE and measurable airborne nicotine was low (kappa = 0.09) (Table 3). Of 86 participants with measurable apartment nicotine, 46.5% were concordantly classified as exposed (i.e. sensitivity or true positives) based on the daily diary, leaving 53.5% discordantly classified (i.e. false negatives). Median measurable nicotine levels among the true positives and false negatives were the same (0.042 μg/m^3^) but the maximum was higher for true positives than false negatives (1.34 vs 0.43 μg/m^3^) (Table 3). Among the 75 individuals with non-detectable apartment nicotine, 62.7% concordantly reported no household TSE (i.e. specificity or true negatives) while 37.3% without measurable apartment nicotine reported TSE in the home.

Spearman correlations showed no association between apartment nicotine concentration (μg/m^3^) and the number of days someone smoked in the apartment nor smelling smoke in the apartment (data not shown). In logistic regression models, we found no associations between anyone smoking in the home or smelling smoke in the home (yes/no) and measurable apartment nicotine (Table 4).

## Discussion

Based on the findings of the Surgeon General, it is commonly accepted that there is no safe level of TSE. This is a basis for surveillance efforts that focus on identifying people and locations experiencing detectable levels of TSE. The Department of Housing and Urban Development’s recent efforts to prohibit smoking in public housing authorities further underscores the importance of understanding the prevalence of TSE in public MUH. In a sample of non-smoking public housing residents living in MUH, we investigated the validity of self-reported TSE as compared to objectively measured personal (saliva cotinine) and household (air nicotine) TSE, which are detectable at extremely low levels. We found poor agreement between self-reported TSE and both measurable cotinine and nicotine. Sensitivity of the overall indicator of self-reported TSE was 68.9% relative to measurable cotinine, leaving 31.1% of those with measurable cotinine classified as unexposed via self-reports. At the same time, specificity was low, and 62.9% of those without measurable cotinine reported TSE. Should self-reported TSE be used as a measure of a smoke-free policy’s success, it might incorrectly indicate a failed policy. For self-reported household exposures using the daily diary, sensitivity was even lower, with only 46.5% reporting TSE in the home among those with positive TSE according to the air nicotine gold standard, leaving more than half of apartments with measurable nicotine classified as unexposed based on resident self-report.

Measured levels of cotinine and nicotine were generally low in our sample, with median detectable levels just over the LOD, and lower than in previous reports in non-smoking residents of MUH [11, 12]. Given the Surgeon General’s indication that there is no safe level of TSE [2, 19], identifying even slightly measurable levels of cotinine or nicotine is valuable for protecting health. Because levels of TSE were relatively low in our sample, individuals may not have been able to accurately recognize or recall TSE. Individuals experiencing low-level chronic exposures may become desensitized to TSE. Indeed, increasing the cut point for measurable cotinine from 0.02 ng/ml to the 75^th^ and 90^th^ percentiles of cotinine (0.06 and 0.15 ng/ml) increased sensitivity of self-reported TSE from 68.9% to 72.2% and 75%, respectively, suggesting better performance of self-reports of TSE at high levels of TSE. Conversely, some participants may be more sensitive to low levels of TSE, and report TSE that is too low to be detected in saliva cotinine. Fully 41.3% of those who reported TSE did not have measurable cotinine. Among those, 89.7% reported TSE in public spaces and 61.5% in private spaces, which suggests that more transient exposures experienced in public spaces are less likely captured in saliva cotinine.

There may be other potential reasons for the poor agreement between the summary self-reported TSE index and measurable cotinine. One may be that our survey did not capture all potential sources of TSE outside of the home. In addition, cotinine has a half-life of about 17 hours [18], and thus exposure that occurred in the distal period of the 7 days may not have been captured in saliva cotinine if exposure was not constant, or did not occur in the latter period. Also, though respondents were asked to report TSE that occurred in the past 7 days, they may respond based on a more general experience outside of that timeframe (telescoping bias).

Reasons for the poor agreement between self-reported household TSE and measurable apartment nicotine may also be due to individuals not realizing they are exposed. Very few reported that anybody had smoked in the home, and it is possible that other household odors from cooking, scented candles, incense, or other factors prevented participants with measurable nicotine in the apartment from detecting the smell of tobacco smoke in the home, especially at low levels. It is also possible that some individuals failed to complete the daily diary appropriately for each day.

While a number of studies have previously compared self-reported measures of duration and intensity of TSE to objective measures of TSE in various settings [24], few studies have compared self-reported dichotomous TSE status (yes/no) to measurable cotinine in adults. O’Connor et al. studied 282 non-smoking pregnant women and found that self-reported TSE exposure based on the criteria “Exposed to someone else’s smoking for at least one hour during the monitoring week” poorly agreed with measurable cotinine in urine samples (kappa = 0.08), and had low sensitivity and specificity (56.0% and 51.8%, respectively), as in our study [25]. Data from a study of more than 1,000 non-smoking adults also found poor agreement between daily self-reported TSE exposure and measurable serum cotinine (kappa = 0.13–15) [26]. Sensitivity and specificity were not reported. In children however, agreement between self-reports (from parents and/or children) and measurable saliva cotinine has been shown to be higher (kappa = 0.47), with a high sensitivity and specificity (85% and 90% respectively) [27]. In a more recent study of cardiology patients, 13 self-reported items were compared to serum cotinine. As in our study, participants most commonly reported TSE exposure in public spaces, and while exposure in the home, car, and other people’s homes correlated with cotinine, exposure in public spaces did not. Sensitivity was low for all self-reported measures and combined indices when compared to detectable cotinine, but increased as the cotinine level increased [28].

Previous studies of the validity of self-reported TSE in pregnant women and children also reported low sensitivity, specificity and agreement with measurable airborne nicotine as in our study. Among pregnant women, comparing TSE status based on a single item (“Exposed to someone else’s smoking for at least one hour during the monitoring week”) against measurable air nicotine measurements in the home, the kappa was 0.29, sensitivity = 51.9% and specificity = 77.0%.[25] Among children, parental reports of TSE using questionnaire items that reported smoking during the measurement period yielded a sensitivity of 61.4% and specificity of 95.7%, while a daily diary of smoking during the measurement period yielded a sensitivity of 55.3% and specificity of 98.0% [29].

### Limitations

Our study population only included non-smoking households, which may affect the generalizability of our findings to settings with household smokers, where TSE would be higher and self-reports more likely to correlate with cotinine. Further, as our questions were designed for ease of administration and interpretation by participants, the self-reported assessments of TSE lacked detail on intensity (e.g. number of cigarettes) and duration (minutes of exposure), which likely limited our ability characterize TSE in ways that better correlate with cotinine and nicotine levels. Previous studies assessing the validity of self-reported TSE against nicotine showed good agreement when the number of cigarettes to which the subject was exposed was ascertained, though such measures reflect higher levels of exposure [30, 31]. Duration and intensity of exposure among adults also showed better agreement than in our study though it was still poor to moderate [26, 32, 33]. Further research should focus on the development of more sensitive measures of self-reported TSE, which may include mapping survey items to objective TSE measures that detail the precise timing and intensity of exposure. Furthermore, as policies increasingly reduce exposures in traditional settings such as in residences, work places, and restaurants, and increase exposure in other settings such as outdoors, it is important to identify and track changing sources of exposure [34]. Lastly, tobacco smoke has thousands of chemicals, including hundreds of harmful constituents. We tracked the same two components of tobacco smoke that have been measured in prior studies so that our results are comparable. While the expectation is that these components diffuse similarly to other tobacco smoke components, and are thus reasonable proxies for the full spectrum of toxicants in tobacco smoke that may travel between spaces in multiunit housing, it is possible that there are important components of tobacco smoke that are detectable by human senses and are not reflected in these measures.

## Conclusions

Our data suggest that self-assessment of TSE alone in non-smoking low-income public housing residents is not adequate to identify individuals with TSE, as 31% with measurable cotinine and 53% with measurable nicotine did not report TSE. As assessments suggest there is no safe level of TSE [2, 19], being able to determine and eliminate TSE even at extremely low levels is important for health. Tobacco-free policies increasingly implemented in MUH, including the proposed national smoke-free policy for public housing authorities are an important and critical step to this end for low-income residents, however the efficacy of these policies need to be continuously evaluated. Self-reported TSE appears to be unreliable for the purposes of policy evaluation because of the high false positive rate. Future studies to identify the burden of TSE in this population should include objective measures, and self-report surveys should focus on private/indoor spaces when weighing the impact of TSE. Understanding the relationship between self-reports and objective measures of TSE can aid in the interpretation of self-reported TSE.
